# Social Value Orientation and Endorsement of Horizontal and Vertical Individualism and Collectivism: An Exploratory Study Comparing Individuals From North America and South Korea

**DOI:** 10.3389/fpsyg.2018.02262

**Published:** 2018-11-20

**Authors:** Chanki Moon, Giovanni A. Travaglino, Ayse K. Uskul

**Affiliations:** ^1^Department of Psychology, Chonnam National University, Gwangju, South Korea; ^2^School of Psychology, University of Kent, Canterbury, United Kingdom; ^3^School of Humanities and Social Science, The Chinese University of Hong Kong, Shenzhen, China

**Keywords:** cultural values, horizontal and vertical individualism and collectivism, social value orientation, cross-cultural comparison, prosocial, proself

## Abstract

Individuals’ cultural tendencies of horizontal/vertical individualism and collectivism interact with their dispositional traits and contextual factors to shape social interactions. A key dispositional trait is social value orientation (SVO), a general tendency toward competition (proself) vs. cooperation (prosocial) in social exchanges. The present study (*N* = 1032) explored the relationship between SVO and personal cultural tendencies of horizontal/vertical individualism and collectivism in two different cultural settings, the United States (a vertical individualist setting) and South Korea (a vertical collectivistic setting). We hypothesized that each value orientation would be associated with the congruent personal cultural tendency across settings. We further hypothesized that this association would be specific to the context, so that SVO would play a more relevant role where the cultural theme was less dominant. Results indicated that, across contexts, proself individuals endorsed vertical individualistic values more strongly than prosocial individuals. Conversely, prosocial individuals endorsed horizontal collectivistic values more strongly than proself individuals. In addition, the effect of SVO was different in the two cultural contexts. Compared to proself individuals, prosocial individuals endorsed horizontal collectivism more strongly in the United States context, and horizontal individualism less strongly in the Korea context. Theoretical implications and limitations of the findings, as well as directions for future work are discussed.

## Introduction

In its broadest sense, culture refers to a system of *shared* meanings, beliefs, and practices that shape the way in which individuals experience and interface with their social and physical environments (Triandis, 2001). Culture provides individuals who belong to the same society or group with a shared template that enables them to interpret reality in similar ways, and interact with each other on the basis of common assumptions (Rohner, 1984).

Importantly, by itself culture does not determine how individuals behave toward others because not everyone adheres to cultural values in the same way (Oyserman et al., 2002). In this paper, we use the label of *cultural values* to indicate the values held at the group level, and the label of *personal cultural tendencies* to indicate individuals’ endorsement of such values. Cultural values indicate the shared standards and the primary modes that may characterize a given group (e.g., a society) at a given time. Cultural values emerge from the institutional, symbolic, and collective arrangements of a society (see Kitayama, 2002). Conversely, personal cultural tendencies refer to individual variability on those standards (Triandis et al., 1995; Triandis, 1996; Fischer, 2006). Thus, cultural values do not have a *necessary* relationship with personal cultural tendencies (Na et al., 2010). Indeed, individuals within the same cultural setting can vary widely on the basis of dispositional traits or due to contextual factors (Mendoza-Denton and Mischel, 2007; Leung and Cohen, 2011).

A key dispositional trait that contributes to explaining how individuals interact with others is *social value orientation* (SVO; Van Lange, 2000). SVO refers to individuals’ preference for competition or cooperation in interpersonal exchanges. Research has yet to investigate the association between SVO and personal cultural tendencies. This is an important research question because it can inform us on how individuals’ cultural inclinations relate to their cooperative/competitive tendencies. In this article, we explore the association between SVO and individuals’ endorsement of horizontal/vertical individualistic and collectivistic personal cultural tendencies in two settings characterized by vertical-individualistic (United States) and vertical collectivistic (South Korea) cultural values (Shavitt et al., 2011a).

## Horizontal and Vertical Individualism and Collectivism

Individualism and collectivism are two broad dimensions which reflect the relative value cultural groups place on an independent and autonomous self, or on the harmony of the group, respectively (Triandis and Gelfand, 1998; Triandis, 2001, 2004). In individualistic societies, people operate in a cultural frame that make them more likely to prioritize their own goals and rely less on contextual factors when appraising others. Conversely, in collectivistic societies, people prioritize group-level goals and place more importance on situational factors when appraising others (e.g., Hofstede, 1980; Markus and Kitayama, 1991; Triandis, 1995; Kitayama et al., 2009).

The original individualism–collectivism dichotomy was subsequently expanded to account for the extent to which different cultural settings value equality (i.e., horizontality) vs. hierarchy (i.e., verticality; Singelis et al., 1995; Triandis, 1995; Triandis and Gelfand, 1998). Specifically, individuals in horizontal individualistic (HI) societies are more likely to consider themselves as independent from and equal in status to others. By contrast, those in vertical individualistic societies (VI) are more likely to emphasize the importance of competition and “getting ahead.” Individuals in horizontal collectivistic settings (HC) are more likely to regard themselves as being equal to and interdependent with others, whereas those in vertical collectivistic societies (VC) are more likely to regard respect for authority as a key value (for reviews, see Shavitt et al., 2006, 2011b). This fourfold typology accounts for important differences in social arrangements and interactions within and across cultures (Sivadas et al., 2008).

Importantly, a growing body of evidence suggests that there exist noteworthy variations in personal cultural tendencies also within cultural groups and that different dimensions can co-exist within the same context (Green et al., 2005; Taras et al., 2016; cf. also Triandis, 1993). For instance, research comparing British and South Korean individuals found that participants from South Korea scored higher on items measuring VI than their British counterparts (Park et al., 2008). Similarly, studies comparing Japanese and American participants found that Japanese participants scored higher on individualism and lower on collectivism compared with American participants (Matsumoto et al., 1996, 1997; see Matsumoto, 1999 for a review).

Moreover, a meta-analysis of 83 studies measuring personal cultural tendencies of individualism and collectivism across different settings indicated that the differences in endorsement of individualism between American and Japanese people disappeared when the importance of competition was accounted for (Oyserman et al., 2002). This suggests that Americans and Japanese may not differ in their endorsement of verticality-related values (see also Shavitt et al., 2006). Relatedly, Oyserman et al. (2002) also demonstrated that South Koreans scored higher on measures of collectivism compared to Americans, but only when items about “*relatedness to others*” were included. This pattern was instead reversed when such items were not included in the analysis. Finally, Green et al. (2005) investigated individuals’ endorsement of horizontal/vertical individualistic and collectivistic values in a sample of 2,533 individuals from 20 different nations. They concluded that, whereas countries are characterized by a prevalent dimension, there were also important variations in horizontal/vertical individualism and collectivism in all countries examined.

Thus, in addition to differences across societies, it is important to consider variations in cultural tendencies within cultural groups. Notably, the extent to which individuals endorse or reject cultural values may vary depending on their personality characteristics or dispositional orientations (cf. Leung and Cohen, 2011). In this study, we focus on the construct of SVO and we examine the relationship between individuals’ SVO and their personal cultural tendencies of horizontal and vertical individualism and collectivism in two different cultural contexts, the United States and South Korea.

### Social Value Orientation

Social value orientation is a key interpersonal orientation that drives individuals’ mode of social interaction (Van Lange, 2000). This concept is grounded in interdependence theory and refers broadly to individuals’ preference for competition or cooperation in social exchanges (Kelley and Thibaut, 1978; see also Van Lange et al., 2007b). More formally, SVO is defined as individuals’ preferences for a particular distribution of unspecified outcomes between the self and others (Van Lange, 2000).

Social value orientation is a relatively stable disposition which develops early in life (Van Lange, 2000), although it can be influenced by successive experiences (Van Lange et al., 1997). This is a key construct that shapes individuals’ behavior and attitudes across a broad array of different settings, including decision making, cooperation, charity giving, problem solving, procedural justice, and political ideologies (e.g., Nauta et al., 2002; Smeesters et al., 2003; Van Lange et al., 2007a, 2012; Van Prooijen et al., 2008, van Prooijen et al., 2012; Emonds et al., 2014).

Measures of SVO generally use individuals’ answers to generic allocation questions to assign individuals to one of three different orientations. Such orientations are defined as (i) *prosocial* (enhancing outcomes equally for both the self and another person), (ii) *individualistic* (enhancing outcomes for the self with very little concern for the outcomes of another person), and (iii) *competitive* (enhancing competitive advantage to obtain more outcomes for the self, relative to another person; Messick and McClintock, 1968; Van Lange et al., 1997). In empirical studies, the prosocial orientation is usually contrasted against the combination of both individualistic and competitive orientations (i.e., proself orientation; e.g., Parks, 1994; Van Lange, 1999; De Cremer and Van Lange, 2001; Smeesters et al., 2003; Van Prooijen et al., 2008). Compared to both individualist and competitors, prosocial individuals are concerned with enhancing both joint outcomes and equality in outcomes (i.e., altruism and equality; see Van Lange, 1999 for a theoretical analysis and empirical evidence). As shown in a meta-analysis by Balliet et al. (2009), the differences between individualists and competitors are typically small or have scarce relevance for different outcome variables.

Recently, Lampridis and Papastylianou (2017) investigated the association between individuals’ attitudes toward different types of prosocial behaviors (e.g., altruism, compliant, emotional, public, anonymous, dire) and personal cultural tendencies of individualism and collectivism in a sample of Greek adolescents. They found that collectivism predicted positive attitudes toward prosocial behaviors, whereas individualism was only associated with positive attitudes toward public (i.e., visible) forms of prosocial behavior.

Realo and colleagues examined the association between individualism and collectivism and social capital, an index of cooperation and trust among individuals (Realo and Allik, 2009; Beilmann and Realo, 2012). This research indicates that the association between culture and prosociality (broadly defined as social capital) is multifaceted and complex (cf. also Inglehart and Welzel, 2005; Welzel, 2010). Whereas there is a significant and positive relationship between social capital and individualism at the national level of analysis, at the individual level of analysis, personal cultural tendencies of both individualism and collectivism were associated to individuals’ levels of social capital. Specifically, in a representative Estonian sample, Beilmann and Realo (2012) found that mature self-responsibility, a component of individualism defined as individuals’ sense of being a ‘causally effective agent’ (p. 209), as well as two components of collectivism [companionship (individuals’ relationships with peers) and patriotism (individuals’ dedication to the nation)] were associated to individuals’ level of social capital.

However, these studies only considered individuals’ attitudes toward prosocial behavior, rather than individuals’ own preferences for a behavioral allocation strategy. In addition, these studies did not consider the role of the vertical/horizontal dimensions of individualism and collectivism. In this paper, we extend this line of research by examining the relationship between horizontal–vertical individualism and collectivism in a VI (the United States) and VC (Korea) society.

## Cultural Contexts, Personal Cultural Tendencies, and Individual Dispositions

Societies are characterized by a prevalent and normative cultural theme that may constrain or promote specific emotions, values, or behaviors, what Kitayama and Markus (1999) defined as *cultural affordances* (see also Kitayama, 2002; Kitayama et al., 2006). Cultural affordances are features of the situation that shape the expression of culture at the individual level. In this research, we compare the South Korean and Northern American contexts.

Cross-cultural evidence indicates that South Korea’s prevalent cultural theme fits in the vertical and collectivistic configuration (VC). In Korea, group harmony and deference for authority are key values (Triandis and Gelfand, 1998; Kim and Markus, 1999; Shavitt et al., 2011b). Compared with other societies, in the Korean context relationships among individuals tend to be governed more strongly by the cultural value of hierarchy (Schwartz, 1999). The society is characterized by relatively higher power distance (House et al., 2004). These cultural traits are linked to the theme of vertical collectivism (Shavitt et al., 2011a,b).

By contrast, empirical evidence suggests that the United States’ prevalent cultural theme is compatible with the vertical and individualistic configuration (VI; Shavitt et al., 2006). In the United States, individuals place a strong importance on the values of independence and uniqueness, as well as status and competition (Markus and Kitayama, 1991; Triandis and Gelfand, 1998; Nelson and Shavitt, 2002). For example, Counihan (1992) examined college students’ ideas about eating by investigating food journals. Results indicates students’ adherence to core American values such as self–control and individual choice, as well as hierarchical social relationships, traits linked to the construct of vertical individualism. In addition, compared to other individualistic societies such as Denmark, individuals in the United States are more likely to celebrate achievement and to value personal success (Nelson and Shavitt, 2002).

Relatedly, Shavitt et al. (2011a) analyzed the content of over 1200 advertisements across five countries including Korea and the United States. Advertisements provide important insights about cultural values (Pollay, 1987; Nelson and Shavitt, 2002). In keeping with the hypothesized classification, ads in vertical countries including both Korea and the United States highlighted the importance of status. However, ads in individualistic countries such as the United States placed more emphasis on the benefits of uniqueness compared to ads in collectivistic countries such as Korea. Taken together, this evidence supports the idea that the prevalent cultural themes in the Korea and the United States are VC and VI, respectively.

However, as discussed earlier, the existence of a dominant, normative cultural theme does not preclude individuals from acting on the basis of contrasting norms and values (Kitayama, 2002; Oyserman et al., 2002). For instance, in the Korean society (VC), some people may still endorse values of self-reliance, uniqueness and equality which are related to horizontal individualism. Moreover, in a society such as the United States (VI), some people may still endorse values of cooperation and interdependence related to horizontal collectivism (Travaglino, 2017). Thus, it is important to investigate under which circumstances individuals are more likely to express personal cultural tendencies that run against the cultural normative theme.

Here, we contend when cultural norms are less prevalent or dominant (i.e., *weaker*), individuals’ dispositions play a stronger role in predicting the expression of personal cultural tendencies. This contention is rooted in the distinction made by some authors between “strong” and “weak” situations (Mischel, 1977; Snyder and Ickes, 1985). Strong situations are characterized by clear norms, and a high degree of certainty and structure. For instance, in a VI society such as the United States norms about the expression of achievement and status are clear and explicit. By contrast, weak situations provide fewer clues to guide the expression of values and behaviors. For instance, in the United States, horizontal-collectivistic norms about cooperation and equality may be less clear and explicit. Whereas strong situations enable individuals to form a shared understanding of what is the appropriate behavior or value, weak situations are characterized by more vague expectations.

Interestingly, research indicates that in circumstances when norms are clear and unambiguous the relative relevance of situational factors in driving behavior becomes stronger whereas that of individual dispositions diminishes. By contrast, when norms are ambiguous, the relevance of situational factors weakens and that of the dispositions increases (Van Lange, 2000). For instance, research demonstrates that SVO predicts sacrifice in close relationships, but only when one’s commitment to the relationship is weak. When one’s commitment is strong, SVO is not associated with sacrifice (Van Lange et al., 1997), suggesting that the role of SVO is less relevant when other, stronger contextual factors are already in play. In a similar vein, Reese et al. (2018) presented evidence that competitiveness traits were more relevant for and had stronger impact on behavior in circumstances in which there was more situational ambiguity (i.e., weaker situations). Specifically, the authors presented three studies demonstrating that individuals’ trait-level desire to win was more relevant in *less* or non-competitive situations. Finally, at the cultural level, Gardner et al. (1999) showed that priming an interdependent or dependent self-construal was more effective in altering individuals’ values and judgements when the prime was inconsistent with the prevalent (dominant) cultural theme. This evidence indicates the importance of considering both situational (cultural level) and individual (personal cultural tendencies) level factors.

## The Present Study

In this study, we examine for the first time the association between individuals’ SVO and their endorsement of horizontal and vertical individualism and collectivism in two different cultural contexts. We explore this association using two large non-student samples from the United States and South Korea. We focus on these two groups because past cross-cultural research indicates that the American context has more prevalent and stronger individualistic norms, whereas in the Korean context, there are more prevalent and stronger collectivistic norms (e.g., Triandis and Gelfand, 1998; Kim et al., 2003). Both contexts share a similar emphasis on verticality and status (Shavitt et al., 2011a,b).

There is little empirical research so far that speaks directly to the association between personal cultural tendencies and SVO and none that compared directly the Northern American and South Korean contexts. Thus, given the lack of directly relevant research, the study reported in this article should be seen largely as explorative. However, on the basis of the previous literature, two general hypotheses can be entertained.

First, one hypothesis is that participants’ SVO is associated with the congruent cultural value in each of the two cultural settings. Previous research has demonstrated that proself (individualist and competitive) individuals are more concerned with their own outcomes and behave more competitively, whereas prosocial individuals are more other-oriented and cooperative (for a meta-analysis see Balliet et al., 2009). These two orientations broadly reflect differences between vertical individualism (i.e., competitiveness) and horizontal collectivism (cooperation). Thus, this evidence may imply the existence of an association between SVO and individuals’ endorsement of these personal cultural tendencies. That is, regardless of the specific cultural context, proself individuals in the United States or South Korea may be more likely to endorse vertical individualistic values whereas prosocial individuals should be more likely to endorse horizontal collectivistic values (“*congruence*” *hypothesis*).

A second hypothesis can be derived from the observation that the relevance of SVO to individuals’ values is a function of the strength of the situation (Van Lange, 2000; cf. Mischel, 1977). Specifically, SVO plays a more central role when the situation is weaker, compared to when other stronger contextual factors are in play (Van Lange et al., 1997). Different societies are characterized by a prevalent dimension of cultural values, as well as internal variations in personal cultural tendencies (e.g., Kitayama and Markus, 1999; Green et al., 2005; Taras et al., 2016). The United States context can be conceptualized as having stronger and more prevalent cultural norms of vertical individualism, whereas South Korea may be conceptualized as having relatively stronger and more prevalent cultural norms of vertical collectivism (Shavitt et al., 2006, 2011a,b). Cultural norms that run directly against these hegemonic themes (i.e., HC in the United States and HI in Korea) are likely to be weaker and more ambiguous.

In the context of this study, existing evidence suggests that dispositional SVO should be more strongly associated with personal cultural tendencies when cultural norms are weaker. This is also in line with the argument that it is easier to experimentally induce variation in personal cultural tendencies that are inconsistent with the context’s dominant cultural value (Gardner et al., 1999). Thus, interesting differences between countries may emerge in the role played by SVO. Specifically, in the United States context, prosocial individuals may be more likely to endorse horizontal collectivistic values compared to proself individuals. By contrast, in the Korean context, proself individuals may be more likely to endorse HI values compared to prosocial individuals (“*culture-specific*” *hypothesis*). These two general lines of reasoning are examined in this exploratory study.

## Materials and Methods

### Participants and Procedure

Six hundred thirteen participants from the United States were recruited (343 men, 267 women, and three others; *M*_age_ = 33.39, *SD*_age_ = 11.62; ethnic background consists of 79.3% White/Caucasian, 4.7% African American, 5.1% Hispanic, 8.2% Asian, 0.3% Native American, 2.4% Others) using Qualtrics *via* Prolific Academic (see Peer et al., 2017). Participants were from different States, including California (9.3%), Florida (7.8%), Pennsylvania (6.7%), Texas (6.0%), and New York (5.9%). The other States represented in the sample each accounted for <5% of the total number of participants.

Six hundred one participants from South Korea (356 men, 244 women, and one others; *M*_age_ = 38.36, *SD*_age_ = 14.83) who identified themselves as Korean were recruited using Qualtrics *via* a Korean research institution which currently holds 767,877 panel members. Participants were from different metropolitan cities and provinces, including Gyeongsang (14.5%), Chungcheong (12.0%), Daegu (11.8%), Seoul (11.3%), Jeolla (9.9%), Busan (8.7%), Gyunggi (7.8%), Daejeon (7.8%), and Gwangju (5.0%). The other regions represented in the sample each accounted for <5% of the Korean sample. All scales included for the present study, originally developed in English, were translated into Korean. Back-translation was used to achieve equivalent meanings in the two languages following guidelines by Brislin (1986). The Korean version of the scales was used in South Korea.

Participants were invited to take part to a study on “social relations.” After completing the measures, both American and Korean participants were debriefed in writing, thanked, and compensated for their time. Both the United States and Korean data for the present study were collected in August 2017. A power analysis using G^∗^Power indicates that our study had a sample size adequate to detect a weak effect (*f* < 0.10) at 90% power (α = 0.05; Faul et al., 2009).

### Measures

#### Social Value Orientation (SVO)

Social value orientation was measured by means of the decomposed games measure (Messick and McClintock, 1968; Van Lange et al., 1997, 2007a). This behavioral measure has been demonstrated to have good reliability and validity (see Van Lange et al., 2007b for a discussion). It consists of nine “games” aimed at assigning people to one of three different orientations, *prosocial*, *individualistic*, and *competitive*. Participants are asked to divide a number of points between themselves and another person. To avoid the effect of strategic considerations in decision making, participants are asked to imagine to be paired with another person who they will not meet or interact with in future.

For instance, participants were asked to select one of the following three allocation options: (A) 510 points for self and 510 points for others, (B) 560 points for self and 300 points for others, or (C) 510 points for self and 110 points for others. In this example, option A represents the *prosocial* choice because it produces the greater joint outcome (510 + 510 = 1020) compared with either option B (560 + 300 = 860) or option C (510 + 110 = 620). Option A also produces the smallest differences between outcomes for self and others (510 − 510 = 0) compared with either option B (560 − 300 = 260) or option C (510 − 110 = 400). Option B represents the *individualistic* choice because it produces the greater absolute outcome for self (560 points) compared with the other options. Option C represents the *competitive* choice because it produces the largest outcomes for the self relative to others (400).

As in previous studies, participants were classified as prosocial, individualistic, or competitive if they made at least six or more choices consistent with one of the orientations (see McClintock and Allison, 1989; Van Lange et al., 1997, Van Lange et al., 2007a). On the basis of this criterion, 373 American participants were classified as prosocial (66.0%), 173 as individualistic (30.6%), and 19 as competitive (3.4%); 252 Korean participants were classified as prosocial (54.0%), 143 as individualistic (30.6%), and 72 as competitive (15.4%). This is in line with a recent review on SVO which suggests that most people are classified as cooperators (46%), followed by individualists (38%), and competitors (12%; Au and Kwong, 2004). Forty-eight American participants and 134 Korean participants could not be reliably classified because they made fewer than six consistent choices. The number of unclassified participants can be as high as 15% of the sample so these values are within the range of acceptability (Van Lange et al., 2007a). The remaining sample size (*N* = 1032) affords enough power to detect a small-to-medium effect (*f* = 0.11) at 90% power (α = 0.05).

In line with previous research, we combined individualistic and competitive orientations to form a single category of *proself* because both individualistic and competitive individuals assign a higher number of outcomes to themselves relative to others (egocentric focus; e.g., Parks, 1994; De Cremer and Van Lange, 2001; Smeesters et al., 2003; Van Prooijen et al., 2008; Balliet et al., 2009). In addition, we note that in this sample the number of competitive individuals in the United States is *N* = 19, too small for meaningful comparisons. Thus, the two categories of *prosocial* vs. *proself* were used in the analyses below.

#### Horizontal and Vertical Individualism and Collectivism

To measure horizontal/vertical individualism and collectivism, we adapted Sivadas et al. (2008)’s measure consisting of 14 items assessing four different factors: horizontal individualism (HI) (three items; e.g., *I enjoy being unique and different from others in many ways*), vertical individualism (VI) (three items; e.g., *I enjoy working in situations involving competition with others*), horizontal collectivism (HC) (four items; e.g., *The well-being of my co-workers is important to me*), and vertical collectivism (VC) (four items; e.g., *I would do what would please my family, even if I detested that activity*). Participants answered items using a seven-point Likert scale (1 = *strongly disagree* to 7 = *strongly agree*). The scale has been validated in four contexts characterized by different configurations of horizontality/verticality and individualism and collectivism (Sivadas et al., 2008). Sivadas et al. (2008) provided initial evidence that the scale performed better to alternative, longer measures (e.g., the 16 items and 27 items scales; Singelis et al., 1995; Triandis and Gelfand, 1998).

## Results

### Construct and Metric Equivalence

To demonstrate that the same four cultural dimensions (HI, HC, VI, and VC) exist in both cultural groups, we examined construct equivalence across the two countries (Van de Vijver and Leung, 1997). Tucker’s phi coefficients were computed to quantify the degree of factorial agreement between cultures. The average Tucker’s phi coefficient across all factors was above 0.95 (the score for individual dimensions ranged from 0.93 to 0.98, *SD* = 0.02), indicating that a good cross-cultural equivalence of each items of cultural value orientation (MacCallum et al., 1999; Lorenzo-Seva and ten Berge, 2006).

Because we were interested in testing the relationship between SVO and the four cultural dimensions across groups, we also sought to establish metric equivalence (He and van de Vijver, 2012). Using a confirmatory factor analysis in R with *lavaan* and s*emTools* packages (Rosseel, 2012), we tested metrical equivalence by specifying two different models with different levels of constrain. First, we tested the configural invariance model to examine whether the same patterns of observed and latent constructs could be found across countries. The fit of the model was not adequate, χ^2^(91, *N* = 1214) = 4786.850 *p* < 0.001, CFI = 0.88, RMSEA = 0.082, SRMR = 0.075.

Therefore, we used an exploratory approach to check that the four cultural dimensions (HI, VI, HC, and VC) loaded on the intended factor in each of the two countries. We used maximum likelihood as the method of extraction and oblimin rotation (Costello and Osborne, 2005). The scree-plot indicated a four-factor solution emerged in each country explaining 52% of the variance in the United States sample, and 43% of the variance in the South Korean sample. Similar to a recent study using this scale in the Northern American context (Travaglino, 2017), items assessing each of the four constructs loaded highly on the expected factor, except the item “*My happiness depends very much on the happiness of those around me*” which loaded on VC in the United States sample (rotated factor loading 0.34) rather than HC. Similarly, in the South Korean sample, the item loaded on VC (0.49) rather than HC. In addition, the item “*Children should feel honored if their parents receive a distinguished award*” loaded on HC in the United States sample (rotated factor loadings 0.33) rather than VC. In the South Korean sample, the item loaded on the right factor but with a relatively small coefficient (0.32). To create consistent scales across the two contexts, these two items were dropped in both samples. The reliability of each of the three items subscale is reported in Table 1.

**Table 1:** Correlations among variables, means, standard deviations, and Cronbach’s alpha for study variables separated by country.

Measure	α_US_	α_KOR_	1	2	3	4	5
1. SVO	–	–	–	0.14^∗∗^	−0.03	0.13^∗∗^	−0.05
2. HI	0.79	0.67	−0.01	–	0.06	0.21^∗∗∗^	0.03
3. HC	0.74	0.74	−0.20^∗∗∗^	0.25^∗∗∗^	–	0.24^∗∗∗^	0.40^∗∗∗^
4. VI	0.81	0.71	0.11^∗^	0.20^∗∗∗^	0.06	–	0.33^∗∗∗^
5. VC	0.76	0.58	−0.08	−0.09^∗^	0.30^∗∗∗^	0.14^∗∗∗^	–
*M*_US_ (SD)	–	–	–	5.37 (0.96)	5.67 (0.90)	4.23 (1.38)	3.74 (1.25)
*M*_KOR_ (SD)	–	–	–	4.41 (1.16)	5.04 (0.90)	4.39 (1.13)	4.23 (0.98)

*Correlations between study variables for American participants (N = 613) are presented below the diagonal, and correlations for Korean participants (N = 601) are presented above the diagonal. For all scales, higher scores are indicative of more extreme responding in the direction of the construct assessed. SVO, social value orientation (coded 1 = prosocial and 2 = proself); HI, horizontal individualism; VI, vertical individualism; HC, horizontal collectivism; VC, vertical collectivism. ^∗∗∗^p < 0.001, ^∗∗^p < 0.01, ^∗^p < 0.05.*

The 12-item version of the scale was thus used to test metric equivalence. A CFA across countries showed that this model (configural invariance model) had adequate fit, χ^2^(96, *N* = 1214) = 318.83, *p* < 0.001, CFI = 0.94, RMSEA = 0.062, SRMR = 0.045. This model was then compared to another model where factor loadings where constrained to be equal across countries (i.e., metric equivalence). This model also had adequate fit, χ^2^(104, *N* = 1214) = 324.61, *p* < 0.001, CFI = 0.942, RMSEA = 0.059, SRMR = 0.046. Importantly, the difference between the two models was not significant, Δχ^2^(8) = 5.78, *p* = 0.67, and ΔCFI = 0.001 (Cheung and Rensvold, 2002), indicating that constraining the factor loadings to be equal across countries did not significantly deteriorate the fit of the model. Table 1 presents the correlations between the variables separately for each country.

### Social Value Orientation and Personal Cultural Tendencies

Small differences in degrees of freedom are due to missing data. To test the congruence hypothesis, we first estimated a structural equation model in which SVO (coded 1 = prosocial; 2 = proself), an observed variable, predicted the four cultural dimensions (latent variables) across countries. Gender and age were added as covariates to the model because the two samples were demographically heterogeneous. However, note that results below are virtually unaltered if the two covariates are not added to the model. Data analyses were conducted using the *R* software with the lavaan package (Rosseel, 2012).

The model fit the data adequately. Chi-square was significant, χ^2^(72, *N* = 1026) = 329.26, *p* < 0.001. However, the other indices indicated a well-fitting model, CFI = 0.93, SRMR = 0.05, RMSEA = 0.06 (90% CI [0.053, 0.066], *p* = 0.01). In line with the congruence hypothesis, HC was negatively predicted by SVO, β = −0.17, *SE* = 0.06, *p* < 0.001. Across the two samples, prosocial individuals were more likely to endorse horizontal collectivistic values. By contrast, VI was positively predicted by SVO, β = 0.13, *SE* = 0.07, *p* < 0.001. Proself individuals were more likely to endorse vertical individualistic values. The associations between SVO and HI, β = 0.01, *SE* = 0.07, *p* = 0.94, or VC, β = −0.03, *SE* = 0.09, *p* = 0.42, were non-significant.

**FIGURE 1:**
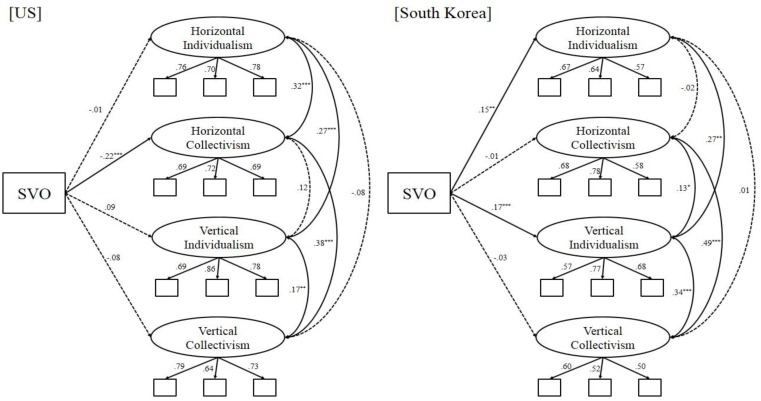
The latent variables model showing coefficients for the predictor of four cultural dimensions (HI, HC, VI, and VC). Dashed lines are non-significant paths. Gender and age are covariates in the model. SVO, social value orientation (coded 1 = prosocial and 2 = proself). ^∗∗∗^*p* < 0.001, ^∗∗^*p* < 0.01, ^∗^*p* < 0.05.

Next, we tested the culture-specific hypothesis using a multi-group approach, as shown in Figure 1. Also, this model fit the data adequately, χ^2^(144, *N*_US_ = 613, *N*_Korea_ = 601) = 426.15, *p* < 0.001, CFI = 0.92, SRMR = 0.05, RMSEA = 0.06 (90% CI [0.055, 0.069], *p* = 0.002). In line with the argument that personal dispositions are more relevant when situational norms are weaker and more ambiguous, in the American sample, the association between HC and SVO was highly significant, β = −0.22, *SE* = 0.09, *p* < 0.001. By contrast, the association between VI and SVO was small and non-significant, β = 0.09, *SE* = 0.11, *p* = 0.052. The difference between the two betas was significant, Δ_(HC-V I)_ = −0.31, *z* = −4.30, *p* < 0.001. Finally, SVO was not significantly associated with HI, β = −0.01, *SE* = 0.10, *p* = 0.84, or VC, β = −0.08, *SE* = 0.13, *p* = 0.10.

In the Korean sample, HI was positively predicted by SVO, β = 0.15, *SE* = 0.10, *p* = 0.006. Conversely, there was no significant association between VC and SVO, β = −0.03, *SE* = 0.10, *p* = 0.63. The difference between the two betas was significant, Δ_(HI-V C)_ = 0.18, *z* = 2.30, *p* = 0.02. There was no significant association between SVO and HC, β = −0.01, *SE* = 0.08, *p* = 0.82. The association between SVO and VI was instead significant, β = 0.17, *SE* = 0.09, *p* < 0.001.

Finally, to examine the differences across the two countries, we compared the multi-group model with other models where the parameters were constrained to be equal across countries. In line with the culture-specific hypothesis, constraining the path between HI and SVO to be equal across countries significantly worsened the model, Δχ^2^(1) = 4.54, *p* = 0.03. When the SVO–HC path was constrained, the two models were also significantly different, Δχ^2^(1) = 8.70, *p* = 0.003. However, when the SVO–VI or SVO–VC paths were constrained across countries, the difference between the two models was not significant, Δχ^2^(1) = 0.21, *p* = 0.64, and Δχ^2^(1) = 0.98, *p* = 0.32, respectively. This pattern of results is in line with the idea that the association between SVO and personal cultural tendencies differ depending on the culture-specific dominant norm. Specifically, SVO plays a stronger role in predicting individuals’ personal cultural tendencies when the country’s cultural value is less prevalent. This pattern of results is also consistent with the idea that the United States and Korea share a similar emphasis on verticality-related norms.

## Discussion

In this study, we explored for the first time the relationship between SVO and individuals’ endorsement of horizontal/vertical individualism/collectivism in two different contexts: the United States (a vertical individualist cultural setting) and South Korea (a vertical collectivistic cultural setting). Drawing on the literature on differences in cultural values and personal cultural tendencies within and across countries (e.g., Green et al., 2005) and dispositional SVO (Van Lange, 2000), we reasoned that individuals’ orientation as proself (more self-oriented) or prosocial (more other-oriented) might be associated with the congruent cultural tendencies of vertical individualism (emphasis on competition) or horizontal collectivism (emphasis on harmony and cooperation), respectively (*congruence hypothesis*).

In addition, we hypothesized that the relationship between SVO and personal cultural tendencies might be different depending on the cultural setting. Previous research demonstrated that the impact of SVO on individuals’ behaviors and attitudes is stronger when situational norms are weaker (Van Lange, 2000; cf. Mischel, 1977). Moreover, previous research has demonstrated that judgements and values are more strongly shifted when individuals are primed with stimuli inconsistent with the context’s dominant cultural value (Gardner et al., 1999). The United States is a vertical individualistic setting whereas South Korea is a vertical collectivistic one. We thus reasoned that SVO would be more strongly associated with individuals’ endorsement of personal cultural tendencies of horizontal collectivism in the United States and of horizontal individualism in Korea because such norms are weaker and more ambiguous in their respective cultural settings (*culture-specific hypothesis*). Results from a large sample size of Northern American and South Korean participants provided supporting evidence for both of these hypotheses.

Specifically, in line with the congruence hypothesis, there was an association between SVO and personal cultural tendencies of horizontal collectivism and vertical individualism. Across the two cultural settings, proself individuals endorsed vertical individualistic values more strongly than did prosocial individuals. By contrast, prosocial individuals endorsed horizontal collectivistic values more strongly than did proself individuals. This result is consistent with the idea that individuals’ SVO is associated with their endorsement of the congruent personal cultural tendencies. To the best of our knowledge, this is the first study to demonstrate this association empirically and it highlights the importance of conducting further research on the relationship between SVO and individual-level cultural tendencies (cf. Beilmann and Realo, 2012).

Notably, the relationship between SVO and personal cultural tendency was moderated by the cultural setting. Specifically, in support of the culture-specific hypothesis, results showed that prosocial individuals in Northern America endorsed HC more strongly than did proself individuals. In South Korea, SVO was not associated with individuals’ endorsement of HC. Conversely, proself individuals in South Korea endorsed HI more strongly than did prosocial individuals. SVO was not associated with HI in the American context.

According to Kitayama and Markus (1999), societies and groups develop a set of institutions, tangible practices and symbolic arrangements that “afford” a dominant cultural theme. These affordances constitute “cultural realities” which play a key role in shaping individuals’ psychological experience of the world (Adams, 2012). Yet, whether individuals embrace such dominant themes is likely to depend – at least partially – on dispositional traits and situational features (see also Triandis, 2001; Leung and Cohen, 2011). Indeed, individuals actively build their social and cultural reality in ways that run against societies cultural prescriptions and shared themes (Adams, 2012). Furthermore, in the specific context of SVO, Van Lange (2000) emphasized the importance of contextual factors in determining the relevance of individuals’ value orientation to a particular behavior or attitude. In line with these propositions, the current findings indicate that SVO is associated with individuals’ endorsement of cultural tendencies, but only when situational norms about the specific value are weaker or more ambiguous (i.e., they run against the culturally dominant theme).

Results also indicated that there was no difference across the two cultural settings in the association between SVO and the vertical dimensions of individualism and collectivism. This result emerged despite the fact that the association between SVO and VI was significant in the Korean sample but not in the Northern American sample. An explanation for the absence of a difference in these two dimensions concerns the fact that there are similarly strong norms about verticality in the United States and Korea. Both settings value competition and hierarchy either at the individual (United States) or at the group (South Korea) level (Shavitt et al., 2011a,b; Cho et al., 2013; cf. also Moon et al., 2018). Because norms are similarly strong across contexts, individuals’ SVO play a similar role in both settings. This explanation is consonant with the observed result indicating that, across the two cultural contexts, proself individuals endorsed vertical individualism more strongly than do prosocial individuals. Moreover, the significant association between SVO and VI in the Korean sample could be due to weaker norms about individualism in Korea (e.g., Moon et al., 2018). Future research should investigate the relationship between SVO and vertical values in different contexts and settings.

### Limitations and Future Directions

This study is the first to investigate the relationship between SVO and personal cultural tendencies in two different contexts. Results provide important new insights about how dispositional orientations toward cooperation and competition and features of the social situation may be associated with individuals’ endorsement of personal cultural tendencies. Nonetheless, this research is affected by some limitations.

First, the scales used to measure horizontal/vertical individualism and collectivism only tapped into general components of these two broad constructs. Research suggests that there are several different ways in which individuals across cultural contexts may value independence or interdependence and that the independence–interdependence binary conceptualization is unable to account for all the possible variations in models of selfhood (see Cross et al., 2011; Taras et al., 2014; Vignoles et al., 2016). For instance, recently Vignoles et al. (2016) identified seven distinct modes of selfhood across cultures. Therefore, an important task for future research is to examine how such modes are associated with individuals’ disposition toward cooperation and competition across different contexts.

Another limitation of this study is that the subscale used to measure VC achieved low reliability in the South Korean sample (α = 0.58). It should be noted that the results from the factor analysis suggested that those items loaded on the same factor and did not crossload on other factors. In addition, the measurement model implemented to test our hypotheses indicated a good fit. Nonetheless, future research should adopt more reliable scales in order to measure vertical collectivism across different contexts.

It should also be highlighted that the SVO measure used in this study resulted in a higher number of non-classified people in the South Korean context relative to the Northern American context (although in both contexts the number of non-classified people was well within the range of acceptability). A possible explanation for this difference is that the measure of SVO used in this study has been developed and employed mainly in Western contexts such as Netherlands or the United States (e.g., Van Lange and Kuhlman, 1994) and may therefore be less suited to detect differences between competitors and cooperators in an Eastern context such as South Korea. For example, according to Chen et al. (2011), a conceptualization of cooperation and competition as two ends of the same continuum may be more prevalent in western cultures, whereas eastern cultures may adopt a more dialectical approach which sees the two allocation strategies equally favorably.

Decomposed games in which participants are asked to allocate resources or select a specific allocation strategy have been used to examine predictions about cooperation and competition in different cultural settings. For instance, research has previously used these games in China (Li et al., 2013; Wei et al., 2016), Bangladesh (Shahrier et al., 2016), Singapore (Balliet et al., 2011), and Japan (Yamagishi et al., 2013; see also Pletzer et al., 2018). Nevertheless, the predictions tested in this research are rooted in the assumption that cooperation and competition stand at the opposite ends of a continuum. This line of research has yet to focus on the important issue of how individuals in South Korea (or East Asia, more generally) represent concepts of cooperation and competition, or what meanings individuals associate with these concepts across cultures and contexts. Future research should address these issues. Future research should also investigate the interplay between competition and cooperation and personal cultural tendencies using different methods, including self-report and experimental methods and comparing different cultural contexts directly. Moreover, future research should also pay more attention to the way in which individuals from different cultural contexts and across different societies conceptualize competition and cooperation.

Finally, an important limitation of this research concerns the fact that only two samples were used to explore the relationship between SVO and personal cultural tendencies. According to the interpretation paradox of cultural differences, differences across widely distinct cultures can be easy to spot but hard to interpret, whereas differences between closely related cultures are harder to find but easier to interpret (van de Vijver and Leung, 2000; Beins, 2011; Boer et al., 2018; Fischer and Poortinga, 2018). The interpretation paradox is a special case of ecological fallacy whereby differences between groups can be mistakenly attributed to cultural factors (Campbell, 1961; see Beins, 2011). With only two cultural groups, it becomes more difficult to determine whether resulting differences in associations are due to cultural level variables or to other factors. Future research should therefore examine the association between SVO and personal cultural tendencies across a wider range and higher number of cultural settings.

It is important to note that, in this research, we did not select cultural clusters at the opposite end of the spectrum. Indeed, both the United States and Korea share the dimension of verticality while differing on the individualism–collectivism axis (Shavitt et al., 2011a,b). This enabled us to explore relatively more specific hypotheses concerning the horizontal dimension of cultural values. Moreover, in this research, we attributed variations across the two contexts not to cultural differences *per se*, but to differences in cultural affordance across setting. Contexts are characterized by dominant values and those values are not always reducible to individual differences (Gardner et al., 1999; Green et al., 2005; Na et al., 2010). Here, we provided initial exploratory evidence for the idea that individual-level dispositions are more strongly associated with personal cultural tendencies that run against the setting’s dominant value. Finally, the two-country comparison used in this paper is compatible with our exploratory approach (see Fischer and Poortinga, 2018, p. 706). This initial evidence should be followed by larger scale cross-cultural studies investigating the interplay between individual dispositions, cultural settings and personal cultural tendencies.

Another promising direction for future research concerns the relationship between SVO and other personal cultural tendencies. For instance, individuals’ SVO may be associated with power distance (Hofstede, 2001; Taras et al., 2012; Daniels and Greguras, 2014) more strongly in more egalitarian cultures compared with less egalitarian cultures. In addition, cultural logics such as honor, face, or dignity may afford different strength to norms of competition and cooperation. Thus, the relevance of SVO for individuals’ endorsement of such cultural logics may vary depending on the specific context.

## Conclusion

Individuals’ endorsement of culturally oriented personal cultural tendencies has important implications for social interactions. However, to understand how cultural tendencies may influence individuals’ social behavior, it is necessary to take into account also individuals’ idiosyncratic dispositions and the relevant features of the social context (Triandis, 2001; Van Lange et al., 2007b). In this article, we explored the relationship between individuals’ SVO and their endorsement of personal cultural tendencies of horizontal/vertical individualism and collectivism. Results support the idea that such orientations play an important role in the way in which individuals endorse (or reject) culturally rooted ideas.

## Ethics Statement

This study was approved by the School of Psychology Ethical Board, University of Kent.

## Author Contributions

CM and GT contributed equally to the manuscript and their names appear in the alphabetic order. CM and GT conceptualized the study idea and performed the analyses. GT drafted the “Introduction” and “Discussion” sections. CM drafted the “Materials and Methods” and “Results” sections. GT revised the manuscript. AU provided critical feedback on both the manuscript and analysis plan.

## Conflict of Interest Statement

The authors declare that the research was conducted in the absence of any commercial or financial relationships that could be construed as a potential conflict of interest.
